# Spoligotype Diversity of *Mycobacterium tuberculosis* over Two Decades from Tiruvallur, South India

**DOI:** 10.1155/2020/8841512

**Published:** 2020-10-14

**Authors:** S. Siva Kumar, S. Ashok Kumar, Gomathi Sekar, K. Devika, M. Bhasker, S. Sriram, C. K. Dolla, Pradeep Aravindan Menon, Srikanth Prasad Tripathy, P. R. Narayanan, Uma Devi Ranganathan, Sujatha Narayanan, Rajesh Mondal

**Affiliations:** ^1^Department of Bacteriology, ICMR-National Institute for Research in Tuberculosis, Chennai, India; ^2^Department of Epidemiology, ICMR-National Institute for Research in Tuberculosis, Chennai, India; ^3^ICMR-National Institute for Research in Tuberculosis, Chennai, India; ^4^Department of Immunology, ICMR-National Institute for Research in Tuberculosis, Chennai, India

## Abstract

Geographically, most tuberculosis (TB) cases in 2018 were reported from India. This TB burden is compounded by MDR-TB and XDR-TB. The strategies for the management and control of TB in the community depend on an understanding of the mode of spread of the different strains of TB isolates in the community. To determine the distribution and trends of *M. tb* strains over the time period in the community due to treatment, we carried out the present study on changes over two decades. *Design/Methods*. A total of 1218  *M. tb* isolates (year: 2001–2018) from Tiruvallur, India, were genotyped by spoligotyping after DNA extraction and subjected to anti-TB drug susceptibility testing for the first-line anti-TB drugs. *Results*. On analysis with the SpolDB4 database, majority (2001–2003: 53.32% and 2015–2018: 46.3%) of the isolates belonged to East African Indian (EAI) lineage, and the orphans designated in comparison to SpolDB4 stood 33% among 2001–2003 strain collection and 46.3% among 2015–2018 strain collection. 10.2% (2001–2003) and 9.26% (2015 to 2018) of isolates were monoresistant to isoniazid (H). MDR strains were less common among EAI strains (3.2%) compared to non-EAI strains (10.32%). *Conclusions*. EAI is the most predominant lineage in Tiruvallur, despite the presence of highly transmissible lineages like Beijing for the last two decades. The prevalence of MDR-TB is below the national average of 2-3% among the new TB cases in the last two decades. The reason can be attributed to the well-established nature of the locally circulating strains in this region which are not associated with drug resistance.

## 1. Introduction

Tuberculosis (TB) is a major cause of morbidity and mortality globally [1]. Data reported by 202 countries and territories showed that 10.0 million people came down with TB in 2018 [1]. Geographically, in the year 2018, the largest number of TB cases were seen in India (27%), followed by those seen in China (9%) [1]. The TB burden is compounded by the presence of multidrug-resistant TB (MDR-TB) and extensively drug-resistant TB (XDR-TB). In 2018, there were about half a million new cases of rifampicin-resistant TB. The largest share of the global burden was in India (27%), China (14%), and the Russian Federation (9%) [1]. Predominant *M. tb* lineages from India and South Asia include EAI, CAS, Beijing, and T lineages [2–7]. A clear difference was observed between *M. tb* lineages circulating in the northern and southern parts of India. CAS predominates in the northern region whereas EAI in the southern part of India [5, 6]. Our hypothesis is that the *M. tb* strains have originated and evolved differently in North and South India, and the host factors would have restricted some strains and had a positive effect on the other, which can be explained by a recent study that describes a sympatric association of host and *M. tb* strains [8]. The other spoligotypes less frequently observed in India are the Haarlem, LAM, S, X, and Manu lineages [2, 3, 6, 7, 9]. Earlier studies have shown that Beijing and Haarlem lineages are associated with first-line drug resistance and XDR-TB from different parts of the world like Vietnam, Taiwan, China, and Russia [10–15]. However, which lineages are more prone to develop resistance in a particular environment has not been well delineated, and there exists a disparity in the reported findings [16].

Resistance to TB drugs and molecular typing of these strains are reported in India from tertiary level hospitals [2, 17], while community-based information is lacking. The ICMR-National Institute for Research in Tuberculosis, Chennai, has been conducting several operational research (OR) activities in Tiruvallur, Tamil Nadu, India, the place where the largest BCG trial was performed [18]. Studies on the molecular epidemiology by our group showed that single and low IS6110 copy strains are most prevalent in this region [3, 19, 20].

The strategies for the management of TB depend on an understanding of the development and spread of TB isolates, but less is known about the changes in the pattern of *M. tb* strains over a period in a community. Long-term trends provide important evidence of relative fitness, but such data are rare. For longer-term trends, a molecular marker with a relatively slow “molecular clock” is required. Spoligotyping provides such a suitable method [21]. Hence, in order to determine the trend of changing spoligotype in the community, we carried out the present study on isolates collected over two decades.

## 2. Material and Methods

### 2.1. Sample Size

The study area Tiruvallur has a population of 580,000 and stretches over 209 villages located about 45 kilometers from Chennai. The samples were collected over a period of 18 years for transmission dynamics at two time points, years 2001–2003 and 2015–2018. Sputum samples were collected from all patients diagnosed with TB and started treatment at any of the government health facilities between January 2001 and December 2003 from the study area. A total of 1110 *M. tb* culture-positive isolates were selected and subjected to genotyping by spoligotyping and antituberculosis drug susceptibility testing. The second set of samples were also collected as part of TB survey at Tiruvallur between the years 2015 and 2018. This study was conducted in the five blocks of the Tiruvallur district of Tamil Nadu, South India. Among the 6340 suspected TB patients, 93.4% (*N* = 5919) sputum samples were collected. Out of a total of 173 *M. tb* culture-positive isolates from the survey, 108 isolates were available for genotyping and antituberculosis drug susceptibility testing. Those diagnosed to have TB were categorized and treated, and their treatment outcome was monitored according to the RNTCP strategy.

## 3. Bacteriological Methods

Sputum samples were transported to NIRT on the same day and were processed by modified Petroff's method for smear by Ziehl–Neelsen staining and cultured on Lowenstein–Jensen (LJ) medium [22]. Anti-TB drug susceptibility testing was performed on cultures positive for *M. tb*. Anti-TB drugs used were Isoniazid (INH), Rifampicin (RIF), Ethambutol (EMB), and Streptomycin (STR) [23, 24]. *M. tb* grown in LJ medium was scraped down into TE (Tris-EDTA) buffer, and the cultures were killed at 80°C before the genomic DNA was isolated by the CTAB-NaCl method as described previously [25]. The genomic DNA extracted was resuspended in TE buffer and stored at −20°C until use.

## 4. Spoligotyping

Amplification of the spacers was accomplished by using the primers DRa (GGTTTTGGGTCTGACGAC) and DRb (CCGAGAGGGGACGGAAAC). All results were entered into Microsoft Excel (version 2003) in a digital format. A binary code of 43 digits was simplified to a 15-digit octal code, which was compared to the updated International Spoligotype Database of the Pasteur Institute of Guadeloupe, which provides information on the shared type distribution of *M. tb* spoligotypes worldwide [26, 27].

### 4.1. Ethical Consideration

This study protocol was approved by the Institutional Ethics Committee of the National Institute for Research in Tuberculosis, Indian Council of Medical Research, Chennai (IEC No: NIRT-IEC-2014032). All diagnosed TB patients were referred to the nearest tuberculosis unit (TU) for further management as per the guidelines.

### 4.2. Results

A total of 1218 *M. tb* isolates (year 2001–2018) were genotyped by spoligotyping, and anti-TB drug susceptibility testing was carried out.

## 5. Spoligotyping

### 5.1. Year 2001 to 2003

**Figure 1:**
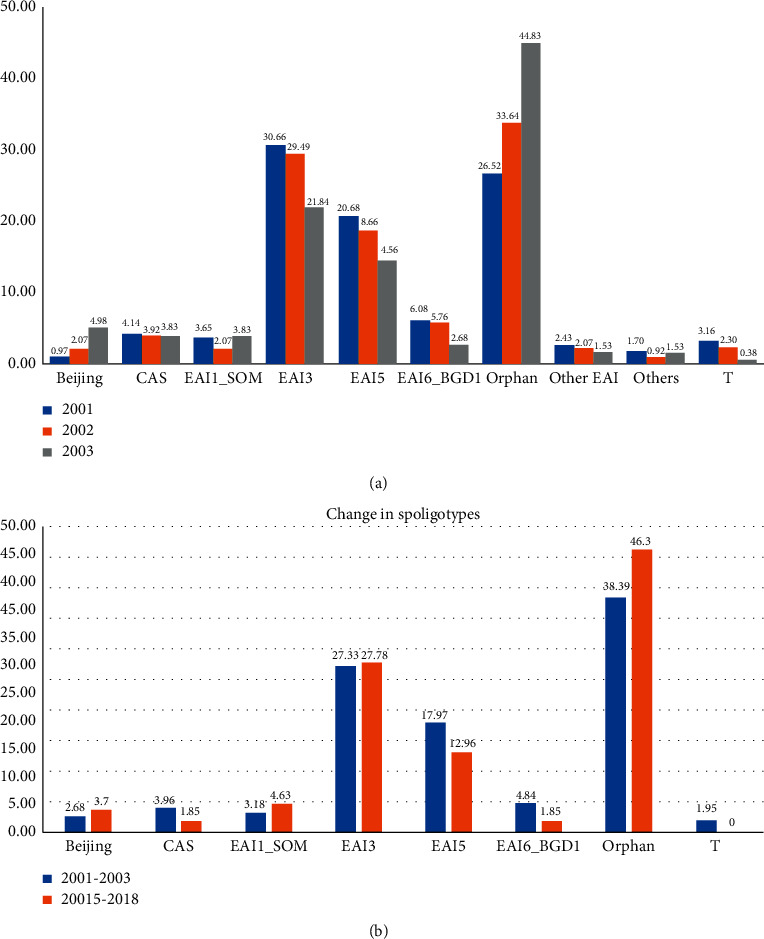
(a) Distribution of spoligotype from the year 2001 to 2003; there is a marked increase in orphan and a decrease in EAI5 and EAI3 strains. (b) Comparison of spoligotype distribution between the years 2001–2003 and 2015–2018; this shows a marked increase in orphan and a decrease in EAI5 strains in this community.

In comparison with the SpolDB4 database, majority (53.32%) of the isolates fit in East African Indian (EAI) lineage, and the orphan strains designated in comparison to SpolDB4 were 38.39%. EAI3_IND is the most common sublineage in this region, followed by EAI5 sublineage. ST 11 (EAI3_IND) was the largest cluster with 26.6% strains, followed by EAI5 ST 340 (6.9%) and EAI5 ST 126 (4.86%). Clustering analysis by spoligotyping exhibits 59.7% clustered isolates. Looking at the year-wise distribution of spoligotypes, there was a considerable increase in orphan strains from 2001 to 2003 (26.52 to 44.83%), which was compensated by a decrease in EAI3 (30.66 to 21.84%) and EAI5 (20.68 to 14.56%) strains. Beijing strains increased from 1% to 5% in three-year time points (Figure 1(a)).

### 5.2. Year 2015 to 2018

**Figure 2:**
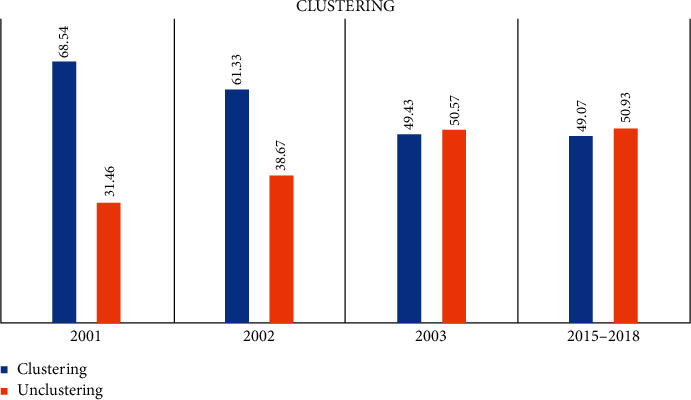
Clustering pattern between the years 2001 and 2018, showing a decrease in clustering, which correlates with an increase in the orphan strains in the community.

**Figure 3:**
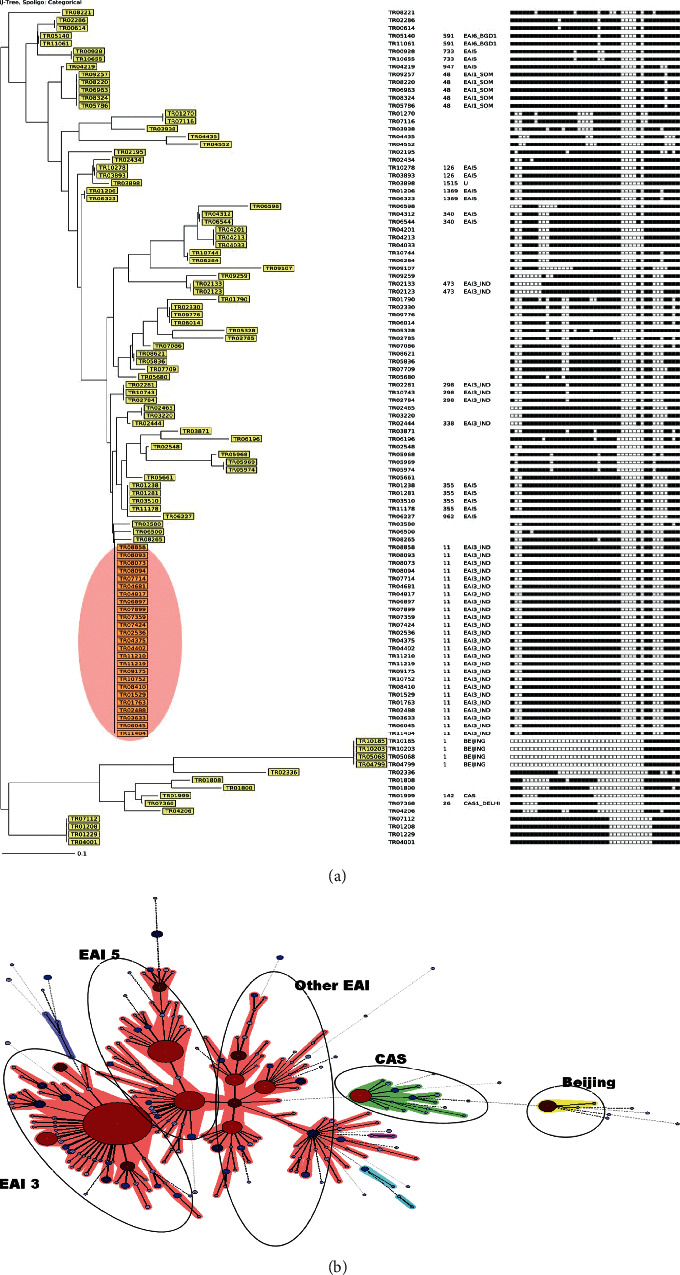
(a) Phylogenetic tree for the recent survey conducted between 2015 and 2018, showing continuous predominance of the EAI3_IND 11 cluster; it also shows the increase in EAI1_SOM 48 strains in this region. (b) A minimum spanning tree (MST) illustrating the distribution of spoligotypes prevalent in Tiruvallur, South India, from 2001 to 2003. The major spoligotypes are labelled against the cluster depicted by the oval. The pink shadow represents East African Indian lineage, green shadow is for Central Asian (CAS) lineage, and the yellow shadow is for Beijing. The structure of the tree is represented as branches and circles representing each individual pattern The colour of the circle is proportional to the number of clinical isolates in our study, illustrating unique isolates as colourless versus coloured clustered isolates (sky blue ≤ 2, deep blue ≤ 5, navy blue ≤ 10, maroon ≤ 20, and red > 20), and also the size of the circle is proportional to the number of strains in the cluster.

Majority (47.2%) of the isolates fit in East African Indian (EAI) lineage, and the orphans designated in comparison to SpolDB4 were 46.3%. EAI3_IND is the most common sublineage in this region (27.78%), followed by EAI5 sublineage (12.9%). ST 11 (EAI3_IND) was the largest cluster with 23.15% strains followed by EAI5 ST 355 (3.7%), followed by other EAI strains. Clustering analysis by spoligotyping exhibits 49.07% clustered isolates (Figure 2). The major cluster was EAI3_IND ST 11 shown by a large red circle (Figure 3).

### 5.3. Year-Wise Distribution of Strains

There was a considerable increase in orphan strains from 2001 to 2018 (26.52 to 44.1%), which was compensated by a decrease in EAI3 (30.66 to 28.8%) and EAI5 (20.68 to 13.5%) strains. Beijing strains increased from 2.68% to 3.7% (Figure 1(b)). Cumulative differences between the data from 2001 to 2003 and 2015–2018 show a marginal increase in the orphan strains and a decrease in EAI5 strains in this survey.

### 5.4. Drug Susceptibility Profile

From the year 2001 to 2003, 10.2% of isolates were monoresistant to INH, one isolate was monoresistant to EMB, and 3.6% were MDR. We observed MDR-TB strains to be less common among EAI strains (0 to 3.2%) compared to non-EAI strains (10.32%). Among the 108 isolates (from the year 2015 to 2018), 11.7% isolates were resistant to INH and two isolates were resistant to RMP. Resistance to any antituberculotic drug was higher among non-EAI (26.3%) strains compared to the EAI strains (18.5%).

## 6. Discussion

Spoligotyping interrogates the DR region of the *M. tb* genome, and the database for the analysis of SpolDB4 has 2740 shared types or spoligotype international types (SIT) containing 53,816 clinical isolates and 4364 orphan patterns [28]. East African Indian (EAI) is the most prevalent lineage in Tiruvallur among both sets of samples from the year 2001 to 2018. Among the isolates analyzed, EAI3_IND ST 11 cluster is the most common cluster found in Tiruvallur, followed by EAI5 by spoligotyping; this has been the trend for the last two decades in this community. The difference between the two sets of samples was the orphan strains (new strains not present in the SPOLDB4 database), which increased from 26.52% in 2001 to 44.1% in the recent survey (2015 to 2018). These were compensated by a decrease in EAI3 and EAI5 strains. At the same time, different survey between 2000 and 2008 in the same region has shown a decrease in culture-positive TB prevalence; this was the region where the first DOTS strategy was initiated in 1999 [29]. It was observed that the prevalence of culture-positive TB was 607, 454, 309, and 388 per 100,000 in the four surveys, and the present study between 2015 and 2018 showed a prevalence of 277 per 100,000 populations [29]. This decrease also correlated with the decrease in clustering in our study.

In India, 2.84% of new TB cases and 11.6% of previously treated cases are estimated to have MDR-TB [30]. India is one of the countries in the world with the highest burden of MDR-TB [1]. The drug resistance also varies among the states of India reported to as high as 7.76% in new and 20% in previously treated patients (drug-resistant survey report, India). In the present study on new TB cases, the EAI strain shows less MDR (1.9%) than the non-EAI (10.3%) MDR strains, and the results show the role of genotypes in the community and their association with drug resistance. In spite of the predominance of EAI lineage in this region, its association with drug resistance has been low compared to the other lineages prevalent elsewhere in India. A report from Pakistan showed that there is absence of correlation between drug resistance and CAS strains (7). However, the present study shows a higher MDR association with non-EAI lineage (CAS and Beijing) compared to EAI lineage, consistent with previous reports from India (2, 17). However, there is no direct relationship between drug resistance and studies have shown that certain genotypes are more prone to drug resistance mechanism; the reasons behind this are yet to be elucidated [31].

*M. tb* spoligotypes have coevolved with their human hosts, giving rise to geographically limited bacterial populations [32]. Even though there has been presence of highly transmissible and drug-resistant Beijing strains [31] in the present community for the last two decades, the transmission has been minimal compared to the EAI strains, and this could be one of the reasons for low drug resistance cases in this region of India; other regions of India where the drug resistance has been reported to be higher demonstrated lower EAI [2, 5, 33]. Some studies suggest that particular lineages of *M. tb* might be adapted to specific human populations and maladapted to others (8). Strain differences in different geographical regions may be linked to different ethnic subpopulations and their migration (15). EAI has been shown to be predominant in Tiruvallur, and it seems well adapted to the study population with low resistance in the last two decades. The clustering rates have also reduced, indicating reduced community transmission, shown by the decrease in the prevalence of TB in this region from 2001 to 2018 (unpublished data).

## 7. Conclusions

EAI is the most predominant lineage in Tiruvallur, despite the presence of highly transmissible lineages like Beijing for the last two decades. There is a marginal increase in the orphan strains but no increase in any resistance or MDR among new TB cases. The reason can be attributed to the well-established nature of the locally circulating strains in this region which are less associated with drug resistance.

## Data Availability

The spoligotyping data used to support the findings of this study are included within the article.
